# Everyday Experiences of Physical Function and Awareness of Fall Risk in Older Adulthood

**DOI:** 10.1093/geroni/igad037

**Published:** 2023-04-26

**Authors:** Shannon T Mejía, Tai-Te Su, Faith C Washington, Sean Golinski, Jacob J Sosnoff

**Affiliations:** Department of Kinesiology and Community Health, College of Applied Health Sciences, University of Illinois Urbana-Champaign, Champaign, Illinois, USA; Department of Kinesiology and Community Health, College of Applied Health Sciences, University of Illinois Urbana-Champaign, Champaign, Illinois, USA; Department of Kinesiology and Community Health, College of Applied Health Sciences, University of Illinois Urbana-Champaign, Champaign, Illinois, USA; Department of Kinesiology and Community Health, College of Applied Health Sciences, University of Illinois Urbana-Champaign, Champaign, Illinois, USA; Department of Physical Therapy, Rehabilitation Science, and Athletic Training, School of Health Professions, Landon Center on Aging, University of Kansas Medical Center, Kansas City, Kansas, USA

**Keywords:** Fall prevention, Microlongitudinal study, Objective and subjective fall risk, Subjective aging, Within-person processes

## Abstract

**Background and Objectives:**

Falls, the leading cause of death and disability among older adults, occur in daily life when the demands of daily activities surpass the ability to maintain balance. An estimated 30% of older adults misestimate their physical function, placing them at greater risk of falling. This study examined how experiences of physical function are linked to awareness of fall risk in daily life.

**Research Design and Methods:**

For 30 consecutive days following a fall-risk assessment, 41 older adults (observations = 1,135; 56% women; age: 65–91) self-assessed objective and subjective fall risk using a custom smartphone application. Alignment of objective and subjective fall risk was indexed as awareness of fall risk. Postural sway was measured by the application. Physical and mobility symptoms and fear of falling were reported daily.

**Results:**

At baseline, 49% of participants misestimated their fall risk. Awareness of fall risk varied from day to day and fall risk was misestimated on 40% of days. Multilevel multinomial models showed individual differences in the level of daily symptoms to increase the tendency to misestimate fall risk. Daily symptoms and fear of falling increased awareness of high fall risk, but daily symptoms threatened awareness of low fall risk.

**Discussion and Implications:**

Findings suggest that misestimation of fall risk is common in older adulthood and informed by appraisals of physical function. Fall prevention strategies could support older adults in understanding their everyday physical function and provide tools to adjust the demands of activities in daily life.

**Translational Significance:** Falls, the leading cause of death and disability among older adults, occur in daily life when activity demands surpass the ability to maintain balance. An estimated 30% of older adults over- or underestimate their physical function, which places them at greater risk of falling. This study examined older adults’ awareness of fall risk in daily life and showed that awareness varies from day to day and is informed by both the accumulation of and day-to-day fluctuations in physical function. Findings urge further research on the development of intervention protocols that are tailored to individualized dynamics of fall risk.

Falls are the leading cause of death and disability among older adults, resulting in ~63% (2.5 million) of nonfatal injuries requiring emergency room treatment and hospitalization (Moreland et al., 2020). Despite decades of focus and scientific inquiry (Gillespie et al., 2012), a previous fall remains the best predictor of a future fall, and the number of fall-related deaths and injuries in older adults continues to increase (Hartholt et al., 2019). Current understanding of fall risk is informed largely by annual clinical assessments of objective and subjective fall risk—the objective functional capacity and subjective appraisal of that capacity to maintain balance. In these annual clinical assessments, ~30% of older adults over- or underestimate their functional capacity (Delbaere et al., 2010). Engagement in daily activities has been linked to subjective experiences on that day (Chi et al., 2021; Jansen et al., 2021). Thus, misestimations of functional capacity could increase fall risk as older adults could either engage in activities that exceed their ability at that moment or become overly cautious and prematurely restrict activity (Fujimoto et al., 2015; Mejia et al., 2023; Sakurai et al., 2013). Although incongruence in clinical assessments of objective and subjective fall risk is common, little is known about how this mismatch manifests as awareness of fall risk in daily life. In this study, we examined older adults’ daily experiences of ­objective and subjective fall risk and physical function over 30 consecutive days. Our purpose was to investigate the extent to which awareness of fall risk varies from day to day and define how daily experiences of physical function contribute to that awareness.

Current knowledge on congruence and incongruence in objective and subjective fall risk—older adults’ *awareness of fall risk*—is informed by annual clinical assessments, which provide single static assessments of objective and subjective dimensions of fall risk (Delbaere et al., 2010; Sakurai et al., 2013). These static assessments provide limited insight into the dynamic nature of functional capacity, older adults’ understanding of their capacity, and exposure to risk (Klenk et al., 2017; Mejia et al., 2023). We propose that the process by which older adults understand and respond to fluctuations in their functional capacity—an *appraisal-action dynamic*—contributes to the occurrence or avoidance of falls in everyday life. Within this dynamic, an older adult would perceive fluctuations in their functional capacity and adjust the demands of daily activities accordingly. However, as older adults commonly misestimate their functional capacity to not fall, the question of how older adults understand fluctuations in their capacity in daily life remains open (Delbaere et al., 2010; Fujimoto et al., 2015; Sakurai et al., 2013). Defining intraindividual processes that drive this everyday awareness of fall risk could inform the development of new prevention protocols and technology-mediated behavioral interventions tailored to individualized fall-risk dynamics.

Day-to-day variation in *awareness of fall risk* could be driven by day-to-day variability in functional capacity and subjective perceptions of that capacity. In concert with older adults’ reports of having “good days” and “bad days,” a growing body of research shows that dimensions of function (e.g., gait, balance, motor control, and reaction time) vary from day to day (Lin & Kelley-Moore, 2017). The magnitude of this intraindividual variability is greater in older populations, suggesting that variability is a property of age-related decline in functional capacity (Lin & Kelley-Moore, 2017). An implication for awareness of fall risk is that these day-to-day fluctuations in functional capacity may be subtle and imperceptible in daily life. Although the amount and diversity of everyday activities decrease in older adulthood (Weber et al., 2020), recent evidence indicates that prosocial behaviors and physical activity vary in concert with appraisals of physical function (Chi et al., 2021; Jansen et al., 2021). Thus, misestimations of functional capacity could lead older adults to engage in activities that either surpass their ability to maintain balance, or alternatively, are not challenging enough to maintain physical function.

Parallel to fluctuations in functional capacity is older adults’ understanding or appraisal of their capacity at a given moment. Subjective experiences of aging, physical symptoms, and self-efficacy exhibit both stable trait-like and dynamic state-like characteristics (Kotter-Grühn et al., 2015; Neupert & Bellingtier, 2017; Zhang et al., 2020). For example, in a study of the oldest old, subjective ratings of functioning were stable over time, despite observed age-related decline in objective measures of functional ability (Wettstein et al., 2016). Research on intraindividual processes has shown somatic symptoms on a given day to be linked to subjective age and sense of control on that day (Barrett & Gumber, 2020; Kotter-Grühn et al., 2015).

Empirical evidence on subjective experiences of health and aging suggests that subjective appraisals of functional capacity at a given moment are formed through momentary experiences and the accumulation of those experiences (Leventhal et al., 2016). For example, dizziness may have distinct relevance for fall risk on a given day, depending on the novelty of the experience and related diagnoses. Changes in physical function have been shown to signal subjective experiences of aging (Miche et al., 2014). However, the implications of day-to-day fluctuations in physical function for awareness of fall risk may differ based on the extent of variation perceived by the individual. Experiences of physical function can range from subtle fluctuations in postural sway and recognition of mobility symptoms to a momentary fear of falling (Hadjistavropoulos et al., 2011). Subtle changes in postural sway may be imperceptible, providing limited implications for awareness of fall risk. Meanwhile, elevated change in physical function could be interpreted as a symptom. Evidence on the link between physical symptoms and control suggests that such symptoms could have implications for falls self-efficacy and thus awareness of fall risk (Zhang et al., 2020). Finally, a change in physical function could elicit a fear of falling—the subjective sense of low self-efficacy in the ability to avoid a fall. However, fear of falling itself may stem from a misestimation of functional capacity, thereby compromising awareness of fall risk (Denkinger et al., 2015; Hadjistavropoulos et al., 2011; Lachman, 2006).

Although falls take place within the context of daily life, the processes by which older adults interpret day-to-day variation in physical function and its implications for functional capacity and appraisal of that capacity are less understood. To move fall prevention forward, our study had two goals. First, we examined the extent of intraindividual variability in awareness of fall risk. As variability in functional capacity is often contrasted with relative stability in subjective experiences of age identity, fluctuations in objective functional capacity were expected to drive variation in awareness of fall risk. Second, we examined individual differences and momentary fluctuations in physical function as mechanisms that inform awareness of fall risk in daily life. We expected fluctuations in physical function to support accurate appraisals of fall risk and experiences more adjacent to falling—mobility symptoms and fear of falling—to be the most salient.

## Method

### Participants

A total of 41 older adults aged 65+ were recruited from the community. The coronavirus disease 2019 (COVID-19) crisis necessitated a shift from in-person to remote baseline assessments. Therefore, the first half of the sample (Panel 1; *n* = 21) was assessed in-person at the fall clinic between October 2018 and May 2020 and the second half (Panel 2; *n* = 20) was assessed remotely via video conferencing by trained fall clinic staff between November 2020 and May 2021. Participants who had wireless internet access, who were able to stand independently for at least 1 min, and rise from a chair without assistance were eligible for inclusion. Panel 1 was restricted to those able to commute to the fall clinic. Panel 2 was restricted to those residing within the same state as the fall clinic. The protocol for the daily portion of the study was equivalent for both panels. Participants received $150 upon completion of the study. The study protocol was approved by the university’s Institutional Review Board.

### Study Procedures

This study utilized an intensive repeated-measures design that included a baseline assessment, 30 consecutive days of self-assessment, and a follow-up assessment. Fall-risk assessment and orientation to study procedures occurred at baseline and took place in the clinic (Panel 1) or via video conferencing (Panel 2). Participants were issued an Android smartphone (Samsung Galaxy S6) outfitted with a daily survey and validated custom application that guided participants through self-assessment of physical function and fall risk (Hsieh et al., 2018). The study protocol was developed to accommodate older adults with little or no experience with either smartphones or remote fall-risk assessment. A custom user manual detailed, with figures and in writing, instructions on smartphone use, survey completion, and the balance task. During orientation, researchers and participants reviewed the user manual and practiced tasks together. Participants interacted with the research team daily during the 30-day study to confirm the successful completion of study tasks and receive technical support as needed. During the daily portion of the study, participants used the smartphone app to (a) assess their subjective fall risk and daily experiences of physical function, and (b) complete a series of tasks to measure postural sway and objective fall risk. The app provided visual and audial cues to guide participants through four balance tasks and one chair-stand task. Each task lasted 30 s and was completed with the smartphone held firmly to the chest. Participants were instructed to wear close-toed shoes, perform tasks on a solid surface with a stable chair within reach, skip a task if they did not feel steady, and indicate on the app if a task was skipped. Researchers observed the first 5 days of the study either in the clinic (Panel 1) or via video conferencing (Panel 2) and contacted participants daily to confirm study protocol compliance.

### Measurements

#### Awareness of fall risk

Accurate awareness of fall risk, the primary study outcome, was conceptualized as the alignment of subjective and objective functional capacity above and below the established fall-risk cutoffs. When objective fall risk was low, awareness of fall risk was indexed as aware of low risk (Q1) or underconfident (Q2), when aligned or misaligned with subjective fall risk, respectively. When objective fall risk was high, awareness of fall risk was indexed as aware of high risk (Q3) or overconfident (Q4), when aligned or misaligned with subjective fall risk, respectively.

**Subjective fall risk** was assessed at baseline and daily during the study using the validated activity-specific balance confidence scale (ABC; Powell & Myers, 1995). The ABC scale is correlated and exchangeable with the Falls-Efficacy Scale (Hadjistavropoulos et al., 2011) and was chosen to minimize the risk of priming participants to consider fear of falling while completing study tasks. The scale was modified for daily assessment. Participants indicated their confidence in maintaining balance while completing common activities *today* (e.g., “bend over and pick up a slipper from the front of a closet floor” or “stand on a chair and reach for something”) by typing a number between 0 and 100 in the text box. Items were averaged to create a balance confidence score at baseline and for each day in the study, with higher values indicating greater confidence. Cronbach’s alpha ranged from 0.94 to 0.97 during the study period, indicating high interitem reliability. Baseline and daily scores below 67 indicated low balance confidence and high subjective fall risk (Lajoie & Gallagher, 2004).

**Objective fall risk** was assessed at baseline and daily during the study using the 30-s chair-stand task, a functional assessment of lower extremity muscle strength and endurance—functions that assist with maintaining balance during daily activities (Jones et al., 1999). Participants were instructed to stand fully from sitting position in a stable chair without assistance as many times as possible within 30 s. Baseline and daily scores that fell below established age- and gender-specific cutoffs indicated high objective fall risk (Center for Disease Control, 2017). At baseline, two researcher-observed trials were completed and the average of the two trials was taken as the baseline score (intraclass correlation coefficient [ICC] = 0.90). During daily assessments, the time series of accelerometry data of the anterior–posterior (*Z*) axis were processed to calculate chair-stand counts (see Supplementary Material Section A). Researcher observations of task performance from the first 5 days of the study were used to validate the accelerometry-based observations. The ICC and area under the receiver-operator curve (AUC) were calculated to assess the agreement in count of sit-stand-sit cycles (ICC = 0.82) and fall-risk classification (AUC = 0.89) between the researcher- and accelerometry-based observations.

#### Daily experiences of physical function

The key independent variables in this study were experiences of physical function.

**Postural sway** was measured during the eyes-open balance task, where participants were instructed by the application to stand for 30 s with their eyes open and feet shoulder-width apart while holding the smartphone firmly to the chest. Postural sway was quantified as the 95% confidence ellipse area (CEA) of the anterior–posterior and mediolateral axes. Test trials conducted with the smartphone app in the lab showed that CEA values greater than 75 would be impossible if the smartphone were held correctly. CEA was set to missing on days that CEA was greater than 75 (4% of observations). See Supplementary Material Section A for further details on the processing of accelerometry data.

**Physical symptoms** were self-reported using a checklist of 13 symptoms developed by Winter et al. (2007). Each day, participants indicated which of the 13 symptoms were experienced at that moment. The count of fatigue, trouble with mobility, dizziness, and muscle stiffness/soreness was calculated to index mobility symptoms. The count of shortness of breath, allergy symptoms, poor appetite, pounding heart, nauseous, forgetfulness, tightness in chest, constipation or diarrhea, and trouble concentrating was calculated to index other physical symptoms.

**Fear of falling** was measured using a single question about fear of falling, which has been validated against the Falls Self-Efficacy scale (Kempen et al., 2008). The question was modified for daily measurement. To avoid priming subsequent performance on functional tasks, participants were asked to report their fear of falling yesterday as follows: “Yesterday, were you worried or afraid of falling?” Responses ranged from “very worried” (3) to “not at all worried” (0). A participant’s answer was taken to represent their fear of falling for the previous day.

#### Covariates

Models were adjusted for awareness of fall risk at baseline. Characteristics of age, gender (1 = man; 0 = woman), and educational attainment (1 = bachelor’s degree; 0 = some college or less) were assessed through a structured questionnaire at baseline.

### Statistical Analysis

To establish intraindividual variability in awareness of fall risk, time-series data of objective and subjective fall risk were visualized and described as the proportion of days spent in each quadrant of awareness of fall risk. Multilevel multinomial logistic regression models, which nest days within persons, were employed to link characteristics of the person and experiences of physical function to the likelihood of the four quadrants of awareness of fall risk. Awareness of fall risk on each day (with awareness of low fall risk serving as the reference group) was regressed on baseline characteristics and awareness of fall risk, the level of experiences of physical function during the study period, and daily experiences of physical function. Time-variant covariates were person centered, allowing coefficients to be interpreted as the association between physical function on a given day and the likelihood of a given quadrant of awareness of fall risk on that day. Time-invariant variables were grand-mean centered allowing the intercept to represent an average person on an average day. As relative comparisons to a single awareness quadrant were difficult to interpret, marginal effects were calculated from the model and reported subsequently. Statistical analyses were performed using STATA 15 (StataCorp, 2017).

## Results

**Figure 1.**
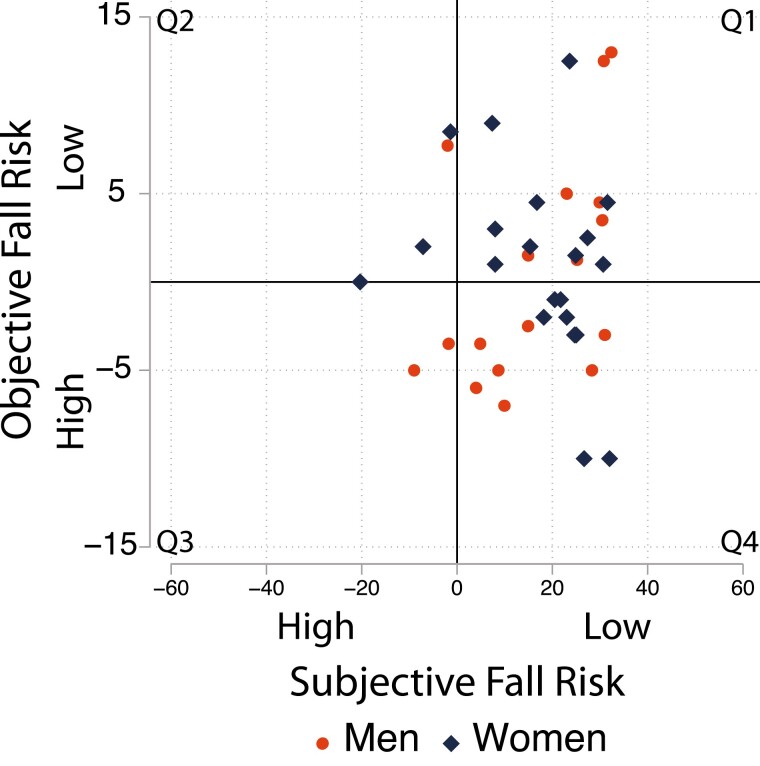
Awareness of fall risk at baseline. Objective fall risk: Baseline chair-stand performance (centered on fall risk cutoffs). Subjective fall risk: Balance Confidence (centered on fall risk cutoffs). Each marker indicates a participants’ objective and subjective fall risk at baseline: Q1: Aware of low risk (*N* = 17; 59% male; age: *M* = 75.93, *SD* = 5.59); Q2: Underconfident (*N* = 4; 75%% male; Age: *M* = 81, *SD* = 7.58); Q3: Aware of high risk (*N* = 4, 25% male; Age: *M* = 76, *SD* = 5.94); Q4: Overconfident (*N* = 16; 56% male; Age: *M* = 72.75, *SD* = 6.52). *SD* = standard deviation.

A total of 41 participants (56% women; 93% identified as White) with a mean age of 75.22 years (standard deviation [*SD*] = 6.75, range = 65–91) completed the 30-day study, providing 1,135 assessments of objective and subjective fall risk. At baseline, 20 participants (50% women) were assessed as high in objective fall risk and 8 participants (50% women) as high in subjective fall risk (Figure 1). The completion rate for the study was high (86%–100% days completed). Negative binomial regression showed missingness to be greater among those without a college degree. Accelerometry data were valid and available in 96% of daily assessments (81%–100% valid observations per person). Differences in baseline participant characteristics across Panels 1 and 2 are presented in Supplementary Table 1. Multivariate regression showed average age (78.62 [±5.75] vs 71.65 [±5.91) and chair-stand performance (13.93 [±5.45] vs 7.47 [±3.45]) to be significantly greater in Panel 1 than Panel 2 (*p*s < .001). Thus, panel membership was maintained as a covariate.

Table 1 summarizes person characteristics, biserial between- and within-person correlations of person characteristics, and the level and day-to-day variation in experiences of physical function. Moderate between-person correlations were observed for chair-stand performance, balance confidence, and postural sway. Those who tended to perform well on the chair-stand task also reported higher balance confidence (*r* = 0.52) and swayed less during the balance task (*r* = −0.42). Similarly, those who tended to report higher balance confidence also swayed less during the balance task (*r* = −0.41), reported fewer mobility symptoms (*r* = −0.46), and less fear of falling (*r* = −0.77). Although average within-person correlations were much smaller in magnitude, a broad distribution was represented in the study sample. Participants differed in how chair-stand performance on a given day was associated with subjective balance confidence (*r* = 0.01, range: −0.55 to 0.83) and postural sway (*r* = 0.07, range: −0.75 to 0.90) on that day. Similarly, differences were observed in how balance confidence on a given day was associated with postural sway on that day (*r* = 0.08, range: −0.57 to 0.52). This diversity in the linear association between day-to-day variation in objective and subjective fall risk suggested that awareness of fall risk—the congruence of objective and subjective risk—would vary across individuals.

**Table 1.** Characteristics of Study Participants and Daily Fall Risk Experiences (*n* = 41, observations = 1,135).

Variable	1	2	3	4	5	6	7	8	9	10	11
Person characteristics											
Age		0.09	−0.03	0.06	−0.22	−0.26	−0.38^*^	0.27	−0.03	0.13	0.20
Male			0.18	0.06	−0.03	−0.21	−0.08	0.33^*^	−0.03	−0.31^*^	0.03
Education				0.11	0.42^*^	0.35^*^	0.43^*^	−0.19	0.01	−0.06	−0.15
BL Chair-stand					0.13	0.50^*^	0.14	−0.29	−0.00	0.01	−0.08
BL Balance Conf						0.56^*^	0.93^*^	−0.45^*^	−0.52^*^	−0.27	−0.77^*^
Daily fall-risk experiences											
Chair-stand							0.52^*^	−0.42^*^	−0.26	−0.03	−0.31
Balance Conf						0.01		−0.41^*^	−0.46^*^	−0.26	−0.77^*^
* min, max*						*−0.55, 0.83*					
Postural Sway						0.07^*^	0.08^*^		0.07	−0.13	0.39^*^
* min, max*						*−0.75, 0.90*	*−0.57, 0.52*				
Mobility Symptoms						−0.08^*^	−0.32^*^	−0.01		0.51^*^	0.57^*^
* min, max*						*−0.52, 0.14*	*−0.84, 0.47*	*−0.54, 0.41*			
Other Symptoms						−0.03	−0.14^*^	0.02	0.23^*^		0.27
* min, max*						*−0.61, 0.56*	*−0.78, 0.43*	*−0.25, 0.47*	*−0.24, 0.95*		
Fear of Falling						−0.01	−0.20^*^	−0.01	0.11^*^	−0.04	
* min, max*						*−0.99, 0.39*	*−0.69, 0.40*	*−0.63, 0.37*	*−0.32, 0.69*	*−0.54, 0.40*	
Descriptive Statistics											
Mean^a^	75.22			11.04	80.40	10.97	79.49	1.88	0.57	0.30	0.40
SD	6.75			5.64	20.15	3.41	21.52	4.00	0.78	0.54	0.49
%		44%	68%								
ICC^b^						0.79	0.94	0.44	0.70	0.65	0.55

*Notes:* Between-person correlations (*n* = 41) presented above the diagonal. Within-person correlations average and range (observations = 1,135) presented below the diagonal. Italics were only used to indicate the range of correlation coefficients. Education: 1 = bachelor’s degree or higher; BL = baseline; balance confidence measured via the Activity-Specific Balance Confidence (ABC) scale; postural sway: confidence ellipse area of eyes-open balance task measured via accelerometry; Mobility and other symptoms: self-reported symptoms checklist; Fear of falling: *t* + 1 of retrospective report of fear of falling yesterday. ICC = intraclass correlation coefficient; *SD* = standard deviation.

^a^Intraindividual mean presented for time-varying covariates.

^b^Intraclass correlation—the proportion of variation attributed to the person. Lower scores indicate greater day-to-day variability.

^*^ *p* < .05.

### Everyday Awareness of Fall Risk

**Figure 2.**
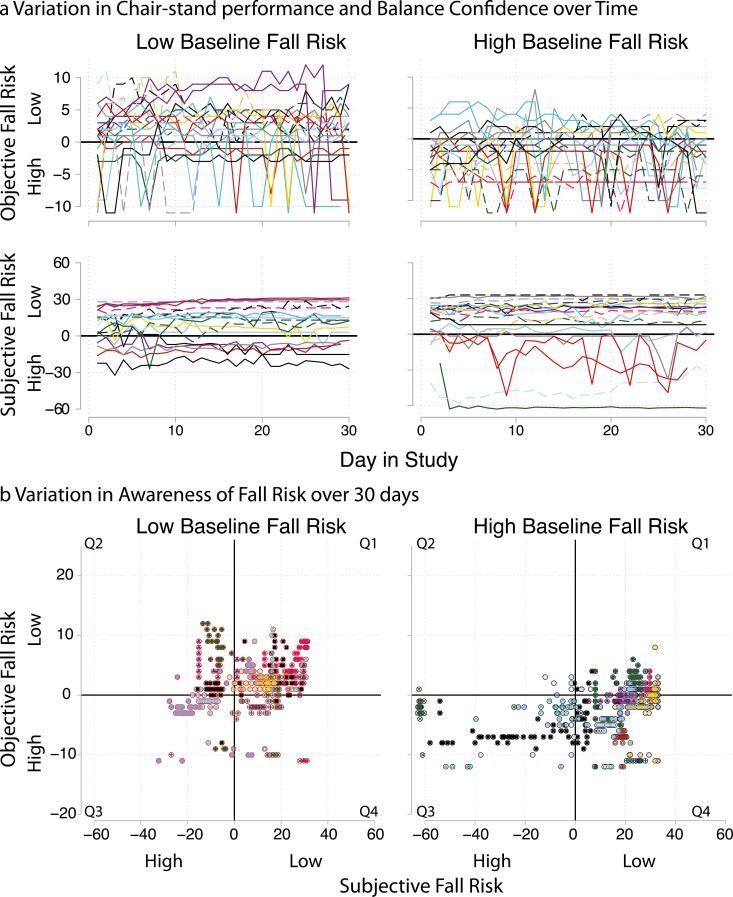
Temporal variation above and below fall-risk cutoffs. Objective fall risk: Daily chair-stand counts (centered on fall-risk cutoffs). Subjective fall risk: Activity-specific balance confidence (centered on fall-risk cutoffs). (A) Each line corresponds with an individual in the study. (B) Each participant has a unique marker style. Each marker indicates an observation of objective and subjective fall risk during the study period. Q1: Aware of low risk (514 obs); Q2: Underconfident (86 obs); Q3: Aware of high risk (163 obs); Q4: Overconfident (372 obs).

Our first task was to establish day-to-day variability in objective and subjective fall risk. As would be expected of validated fall-risk indicators, daily balance confidence and chair-stand performance varied more between- than within-persons, with intraclass correlations of 0.94 and 0.79, respectively. However, as shown in Figure 2A, both chair-stand performance and balance confidence fluctuated above and below fall-risk cutoffs from day to day, even among those assessed as low fall risk at baseline. Figure 2B presents individual differences in how objective fall risk on a given day relates to subjective fall risk on that day and illustrates day-to-day variation in awareness of fall risk. For descriptive purposes, we estimated the person means of the proportion of study days spent in each daily awareness quadrant: 0.45 (range: 0–1) for awareness of low risk (Q1), 0.09 (range: 0.0–0.8) for underconfident (Q2), 0.14 (range: 0–0.97) for awareness of high risk (Q3), and 0.32 (range: 0–1) for overconfident (Q4).

**Figure 3.**
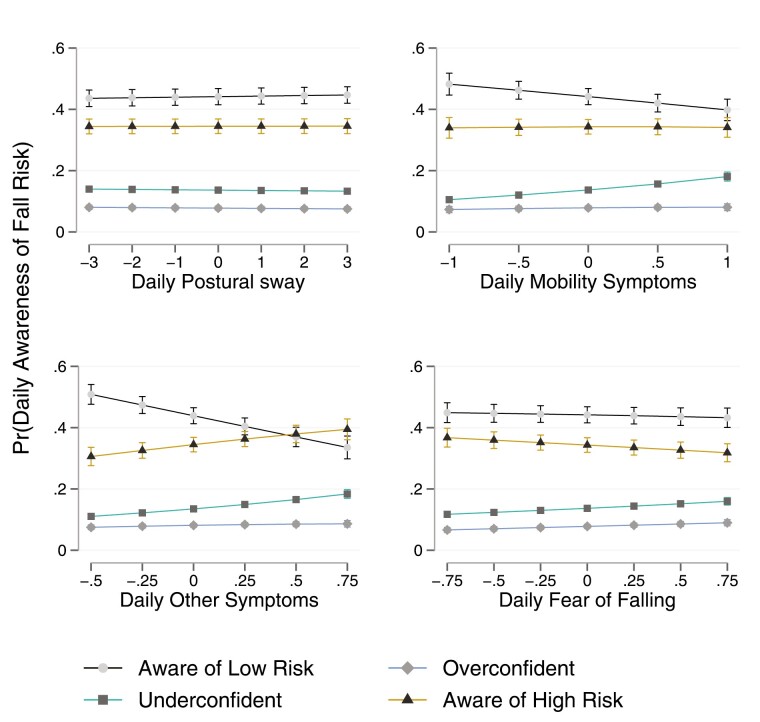
Contributions of daily experiences of physical function to likelihood of daily awareness of fall risk. The figure illustrates how a one-unit increase in the daily experiences of physical function on a given day increases the probability of awareness of fall risk on that day.

After confirming day-to-day variability in awareness of fall risk, we examined how characteristics of the person and experiences of physical function contributed to individual differences and daily variation in awareness of fall risk during the study period. Our multilevel multinomial model distinguished between-person differences in the *general tendency for* from *within-person day-to-day fluctuations in* the alignment of objective fall risk and subjective awareness of that risk. Our focus was on subjective awareness of risk within the context of objective fall risk. As such, two combinations of awareness were of interest: (a) whether participants were underconfident or aware of low risk (Q2 and Q1); and (b) whether participants were overconfident or aware of high risk (Q4 and Q3). Marginal mean probabilities, which show how each covariate contributed to the probability of each awareness of fall-risk quadrant, are reported below and presented in Table 2 and Figure 3. Supplementary Table 2 provides estimates of relative likelihoods from the multilevel multinomial model.

**Table 2.** Contributions of Person Characteristics and Experiences of Physical Function on Predicted Probabilities of Everyday Awareness of Fall Risk (adjusted for study panels).

Variable	Low objective fall risk				High objective fall risk			
	Aware of low risk (Q1 = 514 observations)		Underconfident (Q2 = 86 observations)		Aware of high risk (Q3 = 163 observations)		Overconfident (Q4 = 372 observations)	
	*pr*(Q1)	95% CI	*pr*(Q2)	95% CI	*pr*(Q3)	95% CI	*pr*(Q4)	95% CI
Age	**−0.02**	[−0.04, −0.01]	0.00	[−0.00, 0.00]	**0.01**	[0.00, 0.01]	**0.02**	[0.01, 0.03]
Gender	**−0.22**	[−0.36, −0.09]	0.01	[−0.04, 0.07]	0.04	[−0.01, 0.10]	**0.17**	[0.05, 0.29]
Education	**0.31**	[0.18, 0.43]	−0.02	[−0.08, 0.04]	**−0.17**	[−0.25, −0.09]	−0.11	[−0.24, 0.01]
Study panel	**−0.35**	[−0.56, −0.14]	**−0.10**	[−0.16, −0.05]	0.02	[−0.05, 0.08]	**0.43**	[0.24, 0.63]
Baseline awareness								
Aware low risk (*ref*)								
Underconfident	−0.01	[−0.25, 0.23]	−0.02	[−0.09, 0.05]	**0.30**	[0.16, 0.45]	**−0.27**	[−0.42, −0.12]
Aware high risk	−0.16	[−0.84, 0.53]	−0.05	[−0.17, −0.06]	0.08	[−0.10, 0.25]	0.13	[−0.48, 0.75]
Overconfident	−0.15	[−0.38, 0.07]	0.05	[−0.04, 0.14]	0.02	[−0.05, 0.10]	0.08	[−0.14, 0.30]
Postural sway								
Between-person	0.02	[−0.02, 0.06]	0.01	[0.00, 0.03]	−0.01	[−0.02, 0.00]	−0.02	[−0.05, 0.02]
Within-person	0.00	[−0.00, 0.01]	−0.00	[−0.00, 0.00]	−0.00	[−0.00, 0.00]	−0.00	[−0.00, 0.00]
Mobility symptoms								
Between-person	**−0.24**	[−0.35, −0.13]	−0.01	[−0.04, 0.02]	0.01	[−0.03, 0.06]	**0.23**	[0.14, 0.33]
Within-person	−0.04	[−0.09, 0.00]	0.00	[−0.01, 0.02]	**0.03**	[0.01, 0.05]	0.00	[−0.04, 0.05]
Other symptoms								
Between-person	**0.16**	[0.01, 0.31]	**0.12**	[0.07, 0.17]	**−0.17**	[−0.24, −0.09]	−0.11	[−0.25, 0.03]
Within-person	**−0.14**	[−0.22, −0.06]	0.01	[−0.01, 0.03]	**0.04**	[0.02, 0.06]	**0.09**	[0.02, 0.16]
Fear of falling								
Between-person	−0.05	[−0.26, 0.15]	**0.08**	[0.02, 0.15]	**0.22**	[0.12, 0.31]	**−0.25**	[−0.42, −0.07]
Within-person	−0.01	[−0.06, 0.03]	0.02	[−0.00, 0.03]	**0.03**	[0.00, 0.05]	−0.03	[−0.08, 0.02]

*Notes*: Marginal mean probabilities calculated from multilevel multinomial logistic regression of everyday awareness of fall risk (see Supplementary Table 2). Gender: 1 = man; Education: 1 = Bachelor’s degree or higher; Study Panel: 1 = Panel 2. Values in bold are statistically significant (*p* < .05). CI = confidence interval.

We began with testing how person characteristics differentiated general awareness of fall risk during the study period. Men were less likely to be aware of low risk (*pr*[Q1] = −0.22 [−0.36, −0.09]) and more likely to be overconfident (*pr*[Q4] = 0.17 [0.05, 0.29]). Older age was associated with a decreased probability of awareness of low risk (*pr*[Q1] = −0.02 [−0.04, −0.01]) and an increased probability of both high-risk quadrants (*pr*[Q3] = 0.01 [0.00, 0.01]; *pr*[Q4] = 0.02 [0.01, 0.03]). Those with a college degree were more likely to be aware of low risk (*pr*[Q1] = 0.31 [0.18, 0.43]) but also less likely to be aware of high risk (*pr*[Q3] = −0.17 [−0.25, −0.09]). Panel 2 was less likely than Panel 1 to reside in either of the two low-risk quadrants (*pr*[Q1] = −0.35 [−0.56, −0.14]; *pr*[Q2] = −0.10 [−0.16, −0.05]) and was more likely to be overconfident (*pr*[Q4] = 0.43 [0.24, 0.63]). Baseline awareness of fall risk also differentiated awareness of fall risk during the study period.

After controlling for person characteristics, we examined individual differences in awareness of fall risk across average experiences of physical function. Counterintuitively, those who reported higher-than-average experiences of limited physical function were often less aware of fall risk. In the context of low fall risk ([Q1] aware of low risk; [Q2] underconfidence), the likelihood of awareness of low risk was lower among those who reported more mobility symptoms (*pr*[Q1] = −0.24 [−0.35, −0.13]). Higher-than-average reports of other symptoms, however, were associated with an increased likelihood of both underconfidence and awareness of low risk, suggesting individual differences in how participants with lower fall risk tended to interpret other symptoms; *pr*(Q2) = 0.12 [0.07, 0.17]; *pr*(Q1) = 0.16 [0.01, 0.31]. Consistent with our expectations, a higher level of fear of falling was associated with an increased likelihood of underconfidence during the study period (*pr*[Q2] = 0.08 [0.02, 0.15]).

Meanwhile, in the context of high fall risk ([Q3] aware of high risk; [Q4] overconfidence), a higher level of mobility symptoms was associated with an increased likelihood of overconfidence; *pr*(Q4) = 0.23 [0.14, 0.33]. Similarly, those who reported higher-than-average other symptoms were also less likely to be aware of high risk (*pr*[Q3] = −0.17 [−0.24, −0.09]). In contrast to the other experiences of physical function, those who reported higher-than-average fear of falling were less likely to be overconfident and more likely to be aware of high risk; *pr*(Q4) = −0.25 [−0.42, −0.07]; *pr*(Q3) = 0.22, [0.12, 0.31].

We continued our analysis with an examination of how within-person experiences of physical function on a given day contributed to awareness of fall risk on that day. In the context of low fall risk ([Q1] aware of low risk; [Q2] underconfidence), neither mobility symptoms nor fear of falling on a given day systematically differentiated awareness of fall risk on that day. However, awareness of low risk was less likely on days that more other symptoms were reported; *pr*(Q1) = −0.14 [−0.22, −0.06]. In the context of high fall risk ([Q3] aware of high risk; [Q4] overconfidence), more mobility symptoms and fear of falling on a given day increased the likelihood of awareness of high risk on that day; mobility symptoms: *pr*(Q3) = 0.03 [0.01, 0.05]; fear of falling: *pr*(Q3) = 0.03 [0.00, 0.05]. However, reports of additional other symptoms on a given day increased the probability of both high-risk quadrants on that day; *pr*(Q3) = 0.04 [0.02, 0.06]; *pr*(Q4) = 0.09 [0.02, 0.16]. This suggests that experiences of other symptoms increased the likelihood of high risk on that day, but that an unmeasured property of the day or person differentiated how that risk was interpreted.

## Discussion

As falls occur in everyday life when task demands exceed the capacity to maintain balance, older adults’ understanding of their own functional capacity is essential to fall prevention. In this study, we applied perspectives from subjective aging and appraisals of physiological experiences to understand older adults’ day-to-day awareness of fluctuations in their functional capacity. We observed (a) day-to-day variability in objective and subjective measures of fall risk and awareness of fall risk; (b) differential contributions in the level versus day-to-day experiences of physical function to awareness of fall risk; and (c) the implications of experiences of physical function to increase with its appraised salience for fall risk.

As expected of validated fall-risk indicators, we found both objective and subjective indicators of functional capacity to be relatively stable during the study period, varying more between- than within-persons from day to day. Nevertheless, consistent with research that has documented variability in physical function in older adult populations (Lin & Kelley-Moore, 2017), objective indicators of functional capacity frequently fluctuated above and below fall-risk cutoffs. Consistent with perspectives on resilience of the self in the face of aging (Brandtstädter, 1999) and evidence on daily experiences of awareness of aging (Neupert & Bellingtier, 2017), confidence in the capacity to maintain balance during daily activities varied from day to day, but was relatively stable compared to objective performance on the chair-stand and balance tasks.

This relative day-to-day stability in balance confidence coupled with the observed variability in functional capacity resulted in misappraisals of fall risk in daily life. Indeed, out of the 600 observed days where functional capacity was above fall-risk thresholds, 86% were correctly identified as low fall-risk days. Conversely, of the 535 observed days where functional capacity was below fall-risk thresholds, only 30% were correctly identified as days of increased fall risk. Acknowledging incongruence in the measurement of subjective balance confidence and objective lower extremity strength and endurance, we are confident that the two instruments capture overlapping dimensions of objective and subjective fall risk (Powell & Myers, 1995). Activities within the balance confidence scale, such as walking up or down stairs, walking across the parking lot, and standing on a chair, require lower extremity strength and endurance. Further, in the present study, those with higher balance confidence also performed better on the chair-stand task.

Of central interest in the current study was how momentary experiences of physical function may contribute to awareness of fall risk in daily life. Following dynamic models of risk appraisal (Leventhal et al., 2016), we tested experiences that varied in their explicit association with falling, ranging from subtle fluctuations in the magnitude of postural sway while standing, to reports of mobility and other physical symptoms, and a specific fear of falling. Our approach distinguished between-person differences in the *general tendency for* from *within-person day-to-day fluctuations in* the alignment of objective fall risk and subjective awareness of that risk. The distinction in between- from within-person perspectives on awareness of fall risk and experiences of physical function was found, in this study, to be more than an academic exercise. Broadly, in between-persons, we found that those who reported more symptoms were often less aware of their fall risk. Meanwhile, within-persons, consistent with the proposition that variation from familiar, routine experiences activates awareness of risk (Leventhal et al., 2016), fluctuations in perceptible experiences of physical function, increased awareness of fall risk on that day. In line with research that has shown variation in physical symptoms to signal awareness of aging and age identity (Barrett & Gumber, 2020), increased mobility symptoms and other symptoms were implicated in everyday awareness of fall risk. Echoing calls for a more nuanced understanding of fear of falling (Hadjistavropoulos et al., 2011), in addition to increasing the likelihood of underconfidence, reports of fear of falling were also found to increase awareness of high fall risk and decrease the likelihood of overconfidence.

The implications of our findings are best understood when the circumstances of high versus low objective risk are considered separately. First, a tendency for low objective fall risk required chair-stand scores that were, on average, above fall-risk cutoffs. Consistent with previous research, we found those with greater fear of falling to experience underconfidence more frequently (Delbaere et al., 2010). In this study, underconfidence (or limited awareness of low risk) was also more common among those who tended to report more physical symptoms. Practically, we can imagine these exceptional individuals as acknowledging mobility symptoms in their reports of lower balance confidence, but still performing above fall-risk cutoffs on the chair-stand assessment. This pattern continued within the context of the day, where in the context of low fall risk, other symptoms on a given day were linked to decreased balance confidence on that day and thus threatened awareness of low risk. We note that a higher level of other symptoms was related to a tendency for both awareness of low fall risk and underconfidence. We interpret this counterintuitive finding to indicate the presence of an unmeasured third variable—such as subjective views on aging—that could differentiate how other symptoms are interpreted to affect fall risk (Fundenberger et al., 2022). Taken together, those who are underconfident may misjudge their fall risk because they are more attuned to their momentary mobility. Interventions should focus on developing self-efficacy to limit the risk of activity restriction (Lachman, 2006).

Meanwhile, the tendency to have high objective fall risk, resulting in either awareness of high risk or overconfidence in daily life, would require chair-stand performance, on average, below fall-risk thresholds. Based on previous research, we would expect fear of falling and physical symptoms to facilitate awareness of fall risk (Delbaere et al., 2010). In this sample, overconfidence was commonly observed. Surprisingly, those who reported more physical symptoms were either more likely to be overconfident or less likely to be aware of high risk. Practically, our findings paint a portrait that is consistent with the stability of self within the context of age-related change in physical function (Brandtstädter, 1999; Wettstein et al., 2016). Echoing experimental research that has shown older adults to commonly overestimate their ability (Sakurai et al., 2013), these individuals reported more symptoms, but still tended to rate balance confidence as high while performing below fall-risk cutoffs on the chair-stand task. Indeed, it was only those with higher fear of falling who tended to be aware of their higher fall risk. However, fluctuations in daily symptoms were found to facilitate awareness of higher risk. On a given day, higher reports of physical symptoms or fear of falling increased awareness of higher fall risk on that day. Taken together, our findings suggest that highlighting variation from normal could assist those with higher fall risk to better understand their risk within the context of daily life.

### Implications for Fall Prevention

With the rapid development of mobile sensing technologies, *now is the time* to identify and validate dynamic fall-risk indicators (Hsieh et al., 2023). The findings from this study highlight the importance of subjective appraisals and show how the level and momentary experiences of physical function had distinct implications for awareness of fall risk. New approaches are needed to assess subjective experience with minimal burden. Meanwhile, our findings emphasize the importance of developing dynamic approaches to fall prevention that accommodate day-to-day variability in physical function and individual differences in the interplay of risk indicators (Klenk et al., 2017). Fall prevention that supports awareness of fall risk could leverage mobile technologies to quantify dynamic risk characteristics to tailor fall prevention efforts.

An important next step will be to formally link awareness of fall risk to activity on that day. Physiological experiences and fear of falling on a given day have been shown to alter activity space on that day (Chi et al., 2021; Jansen et al., 2021). We recognize that a portion of daily life attends to obligatory activities that are beyond individual control and difficult to disengage from (Baltes et al., 1990; Weber et al., 2020). Thus, in addition to addressing structural and interpersonal demands, intervention efforts should facilitate awareness and adaptive management of risk so that older adults can engage in activities with confidence. Instruction on an accurate appraisal of functional capacity and strategic adjustment of task demands could reduce the risk of premature activity restriction while also minimizing unnecessary fall risk. Acknowledging that overconfidence may be more adaptive than underconfidence (Delbaere et al., 2010), such training should teach older adults to observe fluctuations in physical function without activating negative thoughts about their own aging (Brothers & Diehl, 2017).

### Limitations and Future Directions

The findings from this study must be viewed within the context of its limitations. First, we note the limitations of our relatively small sample of healthy older adults who volunteered to participate in a study on balance. Differences observed between Panel 1 and the more diverse Panel 2 suggest that higher fall risk and overconfidence are underrepresented in the present sample. Further, this study was designed to examine intraindividual variability in objective and subjective fall risk and daily experiences that may be associated with that risk. The relatively small sample size limited our ability to examine individual differences in these processes. The magnitude of within- and between- person variability in awareness of fall risk and experiences of physical function in this selective sample warrant further investigation in a larger and more representative sample of older adults. Second, the burden of daily assessment required a simplified assessment of objective fall risk. We acknowledge that fall risk is a multidimensional construct and that the assessment of lower extremity strength and endurance provided limited insight into objective fall risk. The ABC was burdensome to participants, and many items were reported as irrelevant to daily life. Further work is needed to develop instruments that assess the nuances of fall risk in daily life. Future work could develop data-driven approaches toward establishing person-centered cutoffs. Finally, this microlongitudinal study offered a correlational cross-section of the intraindividual processes that link awareness of risk to experiences of physical function. Future research should study these processes over time to establish the predictive value of awareness of fall risk for fall prevention.

## Conclusion

This study provides a first and important step toward characterizing the dynamic interplay of daily fall-risk experiences and establishing awareness of fall risk as unique and potentially modifiable fall-risk parameter. We found awareness of fall risk—the congruence in objective functional capacity and subjective appraisal of that capacity—to vary considerably from day to day in a population of healthy older adults. Our findings suggest that older adults’ understanding of their fall risk at a given moment can be informed by appraisals of physical function. Fall prevention should support older adults in understanding their physical function and provide tools to adjust the demands of activities in daily life.

## Supplementary Material

igad037_suppl_Supplementary_Materials
