# Supplementary material for: Everyday Experiences of Physical Function and Awareness of Fall Risk in Older Adulthood
Source: Innov Aging. 2023 Apr 26;7(4):igad037. doi: 10.1093/geroni/igad037 (PMC10237285; doi:10.1093/geroni/igad037)
Supplement: igad037_suppl_Supplementary_Materials [file igad037_suppl_supplementary_materials.docx]

**Online Supplementary Material**

**Section A. Accelerometry Analysis**

Accelerometer data were collected and stored in separate .csv files for each participant while performing each task for each day of the study. Analysis of these .csv files was automated to efficiently determine various postural properties of the participants’ motion while performing these tasks.

Acceleration data from the smartphone in the mediolateral (ML), anteroposterior (AP), and vertical directions were exported, down sampled to 100 Hz using a linear interpolation, and processed with a 4th order, low pass Butterworth filter with a cutoff frequency of 5 Hz. The average acceleration was subtracted from each interpolated acceleration datapoint to center the data. This was repeated separately for the accelerations recorded along each Cartesian axis (x, y, z). Along the y-axis, 9.81 was added to account for the gravitational acceleration.

*Confidence Ellipse Area*

For the x and z-axes, a covariance matrix was calculated and decomposed using singular value decomposition (SVD). The values obtained in the diagonal matrix from SVD, in combination with the inverse cumulative distribution function, were used to find the semi-major and semi-minor axes of the 95% confidence ellipse. The area of the 95% confidence ellipse was computed.

*Chair-stand performance*

Finally, for the fifth round of testing on each day, participant chair-stand (STS) count was calculated by counting the number of peaks present in the participants’ STS acceleration vs. time graphs along the z-axis. To be considered a legitimate instance of standing and sitting, the minimum height of the peak was set to 1.0. This threshold was determined through pilot testing. We performed chair-stands in the lab while holding the phone and compared researcher-counts of chair-stands to various data streams to optimize the algorithm and determine thresholds.

All metadata were exported to a final spreadsheet to easily view participant maximum accelerations and root mean squares along each direction. Accelerometer data were collected and stored in separate .csv files for each participant while performing each task for each day of the study. Analysis of these .csv files was automated to efficiently determine various postural properties of the participants’ motion while performing these tasks.

**Section B: Supplementary** **Table 1.** Comparisons of Participant Characteristics Across Study Panels

|  | Panel 1 (n = 21) | Panel 2 (n = 20) | *p* value |
| --- | --- | --- | --- |
| Person Characteristics | Mean ± SD / N (%) | Mean ± SD / N (%) |  |
| Age | 78.62 ± 5.75 | 71.65 ± 5.91 | <.001*** |
| Male | 11 (52.4) | 7 (35) | .420 |
| Education | 15 (71.4) | 13 (65) | .915 |
| Married | 11 (52.4) | 13 (65) | .615 |
| BL Balance Confidence | 84.59 ± 11.77 | 76.01 ± 25.87 | .176 |
| BL Chair-stand | 13.93 ± 5.45 | 7.47 ± 3.45 | <.001*** |

*Note*. Education: 1 = bachelor’s degree or higher; BL = Baseline; Balance confidence measured via activity-specific balance confidence scale

**Supplementary Table 2**. Multilevel Multinomial Logistic Regression Estimates for the Contribution of Person Characteristics, Level of Experiences, and Daily Experiences on Everyday Awareness of Fall Risk

| Awareness of Fall Risk (ref = low risk, 514 obs) | Underconfident  (86 obs) | | Aware of High-Risk  (163 obs) | | Overconfident  (372 obs) | |
| --- | --- | --- | --- | --- | --- | --- |
|  | *b* | (SE) | *b* | (SE) | *b* | (SE) |
| *Person Characteristics* | | | | |  |  |
| Intercept | -2.55 | (1.66) | -2.17 | (1.61) | 0.83 | (1.24) |
| Day | -0.01 | (0.02) | 0.00 | (0.02) | -0.01 | (0.01) |
| Age | 0.28* | (0.13) | 0.44** | (0.13) | 0.29** | (0.10) |
| Male | 2.80 | (1.44) | 3.63** | (1.24) | 2.84** | (0.97) |
| Education | -5.70*** | (1.49) | -9.10*** | (1.69) | -3.30** | (1.02) |
| Study Panel | -2.01 | (1.97) | 2.40 | (1.95) | 5.51*** | (1.57) |
| Baseline Awareness |  |  |  |  |  |  |
| Low risk (ref) |  |  |  |  |  |  |
| Underconfident | 2.22 | (2.03) | 6.82** | (2.19) | -3.06 | (2.16) |
| High risk | -1.00 | (4.58) | 3.37 | (4.29) | 1.84 | (3.84) |
| Overconfident | 2.92 | (1.69) | 2.64 | (1.73) | 1.54 | (1.26) |
| *Experiences of Physical Function* | | |  |  |  |  |
| BP Postural Sway | 0.04 | (0.34) | -0.33 | (0.32) | -0.28 | (0.27) |
| WP | -0.06 | (0.04) | -0.06 | (0.03) | -0.01 | (0.02) |
| BP Mobility symptoms | 1.51 | (1.08) | 2.48 | (1.11) | 3.36*** | (0.88) |
| WP | 0.95* | (0.42) | 1.60*** | (0.42) | 0.42 | (0.32) |
| BP Other symptoms | 1.92 | (1.40) | -5.86*** | (1.68) | -2.19* | (1.06) |
| WP | 1.92** | (0.61) | 2.79*** | (0.59) | 1.70*** | (0.49) |
| BP Fear of Falling | 7.48*** | (1.71) | 9.28*** | (2.20) | -0.94 | (1.42) |
| WP | 1.18** | (0.42) | 1.23** | (0.46) | -0.07 | (0.32) |
| Variance | 4.25 | (1.62) |  |  |  |  |
| Model Fit | -2LL | (df) | Δ-2LL | (Δdf) | AIC | BIC |
| 1. Person characteristic | 1347.00 | (28) |  |  | 1,403.00 | 1,543.96 |
| 2. Level of Experiences | 1003.35 | (40) | 343.65*** | (12) | 1,083.35 | 1,284.72 |
| 3. Daily Experiences | 926.96 | (52) | 76.37*** | (12) | 1,030.97 | 1,292.76 |
| *Notes*. Estimates from the final, fully adjusted model are presented. BP = between-person; WP = within-person. Education: 1 = Bachelor’s degree or higher; Study Panel: 1 = Panel 2.  ****p* ≤ .001; ** *p* ≤ .01; **p* ≤ .05 | | | | | | |
